# Supplementary material for: Complexome profiling on the Chlamydomonas lpa2 mutant reveals insights into PSII biogenesis and new PSII associated proteins
Source: J Exp Bot. 2021 Aug 26;73(1):245–62. doi: 10.1093/jxb/erab390 (PMC8730698; doi:10.1093/jxb/erab390)
Supplement: erab390_suppl_Supplementary_Dataset_S1 [file erab390_suppl_supplementary_dataset_s1.zip › Supplemental Dataset 1 - Excel List and all profiles/plots/_Cre02.g079400.html]

Trivial name:   
  
Euclidean distance: 10169.77  
Mean Intensity (WT): 1202.63  
Mean Intensity (Mut): 1158.66  
Distance: 8.46  
  
MapMan: RNA.RNA binding  
  
p value of intensity sums Welch test: 0.9605
